# Mapping of temperate upland habitats using high-resolution satellite imagery and machine learning

**DOI:** 10.1007/s10661-024-12998-0

**Published:** 2024-08-31

**Authors:** Charmaine Cruz, Philip M. Perrin, James R. Martin, Jerome O’Connell, Kevin McGuinness, John Connolly

**Affiliations:** 1 Department of Geography, Trinity College Dublin, The University of Dublin, Dublin, Ireland; 2 Botanical, Environmental & Conservation (BEC) Consultants Ltd, Dublin, Ireland; 3 Proveye Limited, Causeway, Co. Kerry Ireland; 4 School of Electronic Engineering, Dublin City University, Dublin, Ireland

**Keywords:** Upland habitats, Crisp and fuzzy classification techniques, Random Forest, Pleiades satellite image

## Abstract

Upland habitats provide vital ecological services, yet they are highly threatened by natural and anthropogenic stressors. Monitoring these vulnerable habitats is fundamental for conservation and involves determining information about their spatial locations and conditions. Remote sensing has evolved as a promising tool to map the distribution of upland habitats in space and time. However, the resolutions of most freely available satellite images (e.g., 10-m resolution for Sentinel-2) may not be sufficient for mapping relatively small features, especially in the heterogeneous landscape—in terms of habitat composition—of uplands. Moreover, the use of traditional remote sensing methods, imposing discrete boundaries between habitats, may not accurately represent upland habitats as they often occur in mosaics and merge with each other. In this context, we used high-resolution (2 m) Pleiades satellite imagery and Random Forest (RF) machine learning to map habitats at two Irish upland sites. Specifically, we investigated the impact of varying spatial resolutions on classification accuracy and proposed a complementary approach to traditional methods for mapping complex upland habitats. Results showed that the accuracy generally improved with finer spatial resolution data, with the highest accuracy values (80.34% and 79.64%) achieved for both sites using the 2-m resolution datasets. The probability maps derived from the RF-based fuzzy classification technique can represent complex mosaics and gradual transitions occurring in upland habitats. The presented approach can potentially enhance our understanding of the spatiotemporal dynamics of habitats over large areas.

## Introduction

Uplands comprise a range of extensive, mostly semi-natural habitats, including blanket bogs, heaths, fens, grasslands and those associated with exposed rocks and scree (Perrin et al., 2009). These habitats are protected in the European Union (EU) under the Habitats Directive (HD) (Commission of the European Communities, 1992). They provide important services, such as carbon sequestration and storage, biodiversity support, flood mitigation and water quality regulation (Bonn et al., 2008). However, they are also highly vulnerable to climate change and to increasing pressures and threats from anthropogenic stressors, mainly by land-use changes (Connolly, 2018; Crowle & McCormack, 2009; Perrin et al., 2017; Young et al., 2005). These stressors can lead to habitat fragmentation and widespread degradation, causing these habitats to lose their capacity to deliver such services (Connolly, 2018; Perrin et al., 2009). In the recent ‘State of Nature in the EU’ report, only 15% of the habitats in the EU were in favourable condition, with blanket bogs showing a deteriorating trend (European Environment Agency, 2020).

The alarming decline in habitat conditions within the EU has been recognised by the European Commission (EC) through the proposed Nature Restoration Law (European Commission, 2022). This law will require EU member states to develop and implement restoration measures for degraded ecosystems and habitats, such as drained bogs. Each member state must also monitor and report its progress in implementing the law (European Council, 2023). Comprehensive mapping is fundamental for executing this legal requirement as it can provide baseline data, such as the location and extent of habitats, and can be used to monitor and track their condition over time. Common methods to map habitats include ground-based field surveys or interpretation from aerial imagery (Smith et al., 2011). However, the vast, remote and rugged terrain of upland habitats may limit the frequency of manual field surveys to conduct mapping of these habitats as there are issues with resource availability (i.e., limited time and cost) (Buchanan et al., 2005; Müller & Brandl, 2009). Using aerial imagery to delineate habitat boundaries—in the form of polygons—can also be challenging as several upland habitats typically occur together as mosaics, making it difficult to represent them as separate polygons (O’Connell et al., 2014; Perrin et al., 2009). A complementary approach to address these mapping challenges should be considered, particularly with the increased need for accurate, timely and broad-scale spatial information on these protected habitats.

Remote sensing is a promising approach to mapping and monitoring habitats and vegetation communities within them (Corbane et al., 2015; Mücher & Hazeu, 2021; Nagendra et al., 2013). Specifically, optical imagery acquired by satellites has been used for mapping upland habitats (Barrett et al., 2016; Connolly, 2018; Mehner et al., 2004; O’Connell et al., 2014). The spatial extent that a single satellite image can cover is a significant advantage, particularly in upland habitats, as they can be extensive (JNCC, 2015). Moreover, satellite remote sensing technologies have significantly improved over the last decade (Cantrell et al., 2021; Gleyzes et al., 2012; Kim et al., 2022). They now provide imagery at higher spatial, spectral and temporal resolutions than previously available (i.e., spatial resolution of a few metres with less than a week revisit time). Previous studies have demonstrated that higher spatial resolution imagery (< 5 m) can improve classification accuracy. However, these studies were mainly focused on general land cover classes wherein spectral characteristics can be quite distinct (Boyle et al., 2014; J. Fisher et al., 2017). The impact of image spatial resolution on classifying spectrally similar habitats, such as in uplands, has not been fully explored.

Most upland habitat mapping studies have used the conventional hard or crisp classification technique (Barrett et al., 2016; Mehner et al., 2004). This technique involves a classifier making a definitive decision on which class a pixel will be assigned to, typically to the class with the highest proportion in that pixel. Each pixel is assumed to be pure; hence, it is only associated with a single class (P. Fisher, 1997). However, information in a satellite image pixel often consists of multiple classes. As some habitats are often intermixed, the crisp classification technique may mean that information about other classes will be omitted (Lucas et al., 2007). Moreover, the technique can result in abrupt transitions between habitats in the maps, which poorly describe the continuous nature of upland habitats. Upland habitats rarely have sharp boundaries. They are mainly heterogeneous, occurring in complex mosaics (Gatis et al., 2022; O’Connell et al., 2014). A soft or fuzzy classification technique can be used to address the challenge of mapping transitional boundaries in the uplands. In contrast to the crisp classification technique, the fuzzy classification technique allows each image pixel to be described by the probabilities of occurrence of all classes being considered (Foody, 1996). This technique can allow the representation of mixed and gradual transitions that often exist between habitats in complex environments, such as in uplands (Feilhauer et al., 2021). Moreover, the area computed from class probabilities can also be more accurate and closer to the actual area of that class (Sales et al., 2022).

Random Forest (RF) is a widely used machine-learning algorithm for classifying habitat and vegetation because of its robustness and excellent results (Amani et al., 2017; Cruz et al., 2023; van Iersel et al., 2018). RF (Breiman, 2001) is an ensemble learning made up of decision trees, wherein each tree makes its own class prediction. The final class prediction has the most votes over all these trees (i.e., majority voting). RF can also provide information on class probabilities (Malley et al., 2012). This attribute of RF, which has been used by previous satellite-based remote sensing studies (O’Connell et al., 2015; Raab et al., 2018; Sales et al., 2022), could be explored for classifying upland habitats.

This study aimed to utilise a combination of machine learning and high-resolution satellite imagery to map complex upland habitats. Specifically, the objectives are the following: (1) to determine the impact of spatial resolution on classification accuracy, (2) to compare crisp and fuzzy classification approaches using the RF algorithm for mapping upland habitats, and (3) to apply RF-based models to map and describe the spatial distributions of habitats at two studied Irish upland sites.

## Materials and methods

### Study sites

**Fig. 1:**
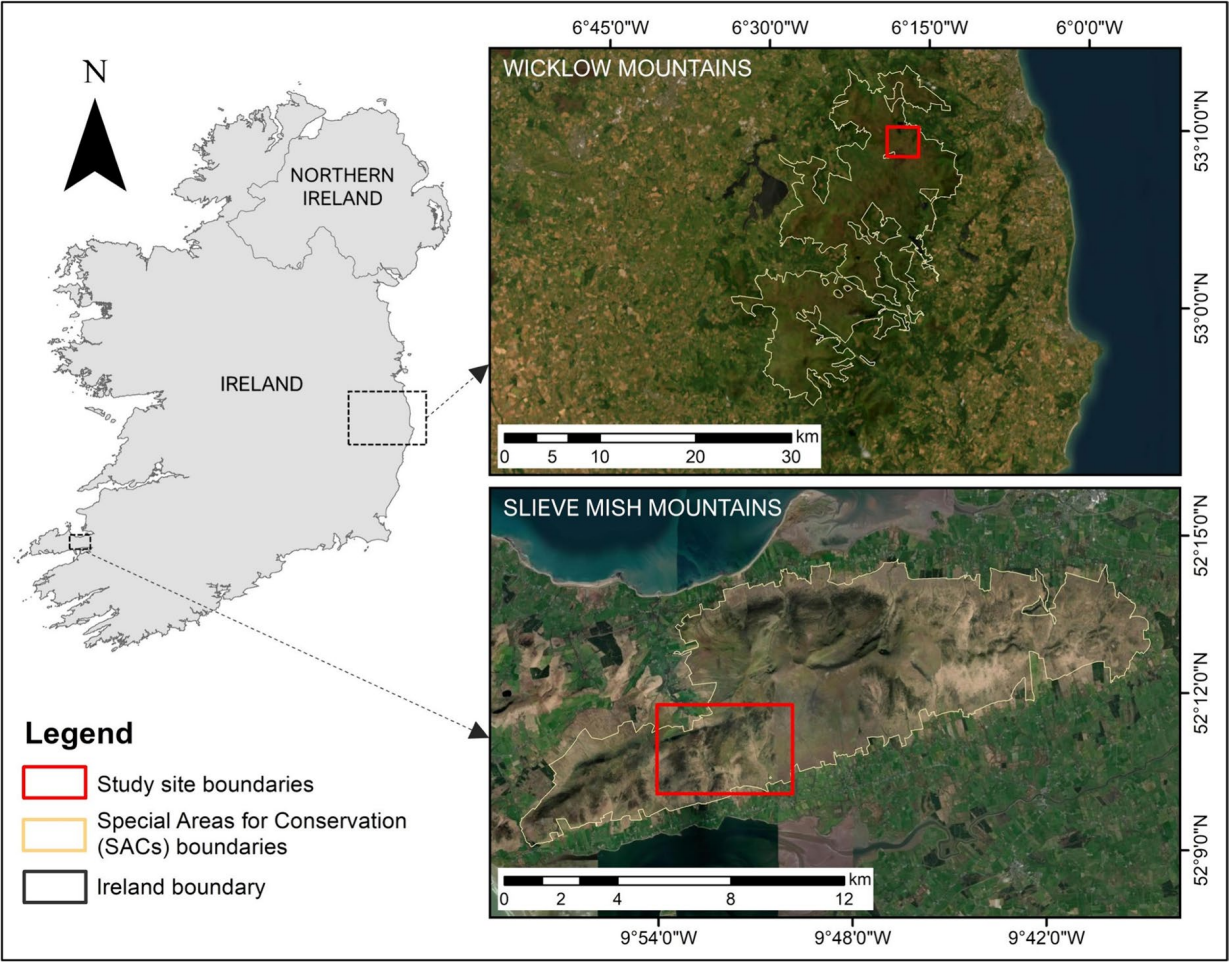
Inset maps showing the locations of the two study sites, Wicklow Mountains (top right) and Slieve Mish Mountains (bottom right), and the boundaries of the SAC covering each site

**Fig. 2:**
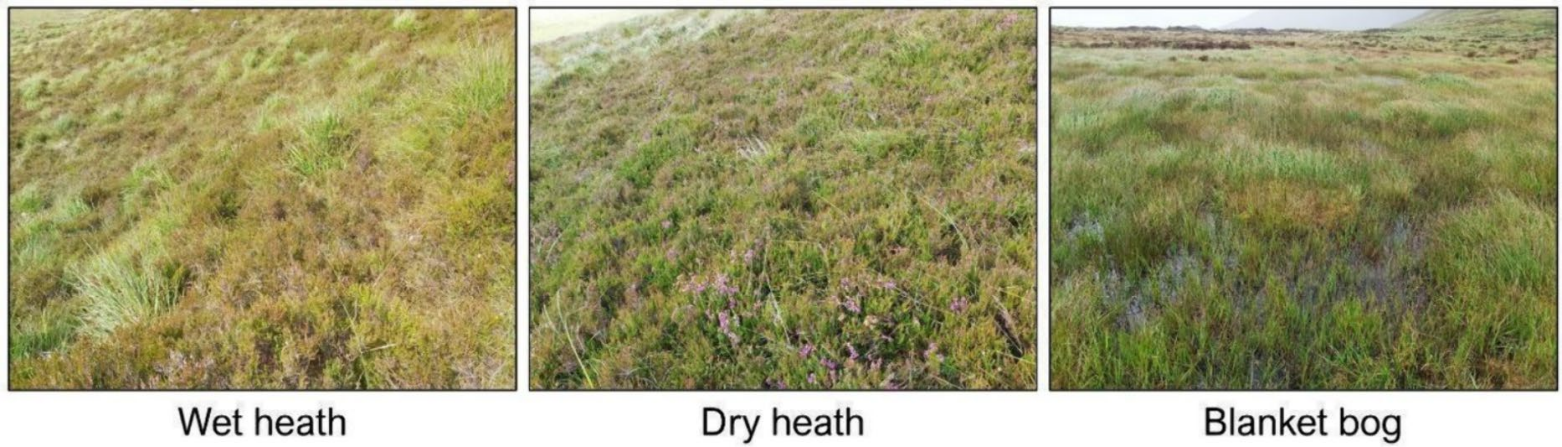
Sample field pictures of the three main upland habitats in the study sites: wet heath, dry heath and blanket bog

Two Irish upland sites, one in the Wicklow Mountains and one in the Slieve Mish Mountains, were selected for this study (Fig. 1). Both sites are designated as Special Areas of Conservations (SACs), part of the Natura 2000 network (National Parks & Wildlife Service, 2016, 2017), the world’s largest network of protected sites covering the most valuable yet threatened habitats and species in the EU territory (Evans, 2012). The two sites are dominated by three upland habitats protected under the HD (those listed under Annex I): wet heath, dry heath and blanket bog (Perrin et al., 2014, 2017) (Fig. 2). The study focused on classifying these three Annex I upland habitats. Other common habitats present at each site were also classified as some of them represent disturbance on uplands, such as the eroding blanket bog and the presence of dense bracken.

The first site (WM) is in the Wicklow Mountains, located south of County Dublin. WM covers an area of approximately 1,020 hectares, which is just over 3% of the entire Wicklow Mountains SAC extent. This site is a good example of a mountain blanket bog in eastern Ireland, consisting of deep peat with depths ranging from one to six metres (Holden & Connolly, 2011).

The second studied site (SM) is in Slieve Mish Mountains SAC, located on the eastern side of the Dingle Peninsula in County Kerry. SM extends to an area of approximately 1,500 hectares, about 15% of the Slieve Mish Mountains SAC extent, which is 9,790 hectares (Perrin et al., 2014).

### General methodology

**Fig. 3:**
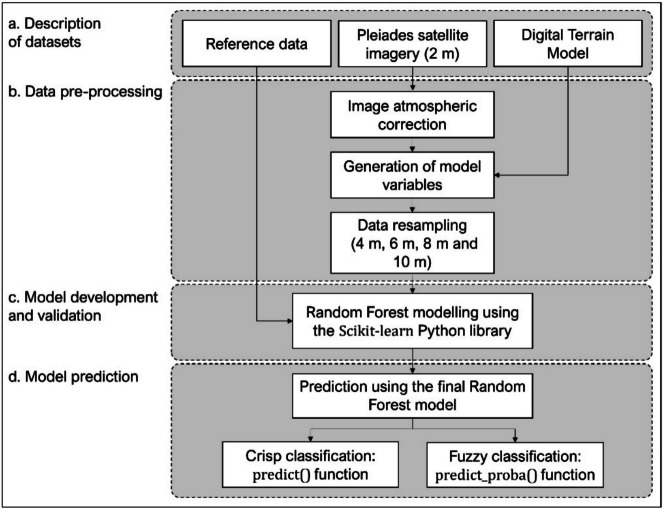
Diagram of the workflow illustrating the processing performed in the study

High-resolution (2 m) Pleiades satellite imagery and Digital Terrain Models (DTMs) were obtained for both sites. Several raster layers, or variables, were generated for each site using the obtained datasets to describe the characteristics of each habitat. These variables were resampled from the original 2 m resolution to 4 m, 6 m, 8 m and 10 m resolutions, generating five sets of variables. A reference dataset was used to train and evaluate an RF-based classification model for each set. The trained model was then applied to produce spatially continuous crisp and fuzzy-classified images within the study site. Figure 3 provides an overview of the workflow.

#### a. Description of datasets

##### Pleiades satellite imagery and Digital Terrain Model

Cloud-free Pleiades images for each site were downloaded from the Sentinel Hub EO browser through the European Space Agency sponsorship program (sentinel-hub.com/Network-of-Resources/). The downloaded images for the SM and WM sites were captured on 15 April 2020 and 8 May 2022, respectively, which closely aligns with the collection period of field datasets. Both images have a spatial resolution of 2 m and consist of four spectral bands on the following wavelengths: blue (430–550 nm), green (490–610 nm), red (600–720 nm) and near-infrared (750–950 nm). The downloaded images were already orthorectified and projected on the Universal Transverse Mercator Zone 29 North coordinate system. DTMs for both sites were also obtained and reprojected to the same coordinate system as the Pleiades images.

To ensure the classification only focused on the upland area, the image extents for both sites were modified to align with the boundary used in the national survey of Irish upland habitats (Perrin et al., 2014). This boundary was based on the definition of upland habitats by Perrin et al. (2009), i.e., any unenclosed land areas found at altitudes above 150 m and contiguous areas of related habitats below this value. The downloaded image for the SM site was clipped using the boundary. No clipping was necessary for the WM site image as its extent was already within the boundary.

##### Reference dataset

For this study, a reference dataset was used that consisted of data collected both in the field and through on-screen digitisation. In the field, the ecologists used Global Navigation Satellite System receivers (centimetre-level accuracy) to record the location of a habitat, represented by a point. To ensure the recording of the correct coordinates of a point within a particular habitat, each point was measured at the centre of an area of a relatively homogeneous habitat cover. Ecologists also recorded the corresponding Annex I habitat name and photographed it in the north-view direction. If a point was not an Annex I habitat, the Fossitt habitat classification scheme was used to label it. Fossitt (2000) provides a standard guide for habitat identification in Ireland. The field data points were then visually inspected on the screen by the remote sensing analyst to ensure they aligned with the correct habitats, as seen on the satellite image. A similar process was applied to the additional points added in a GIS environment to supplement the field data. This process consisted of manual and subjective interpretation of the satellite image guided by the data from previous field surveys (Perrin et al., 2017; Perrin et al., 2014). These previous survey data consist of polygons with records of approximate percentages of the constituent habitat types. Hence, the additional points were based on those polygons mostly comprising a single habitat (> 98%). Table 1 provides the list of habitats considered for each study site. To account for the shaded areas in the image caused by tree canopy and steep slopes, we added a pseudo-habitat ‘shadow’ to the lists (Table 1).

**Table 1:** List of habitats for each study site

Study site	Habitats
WM	Wet heath Dry heath Blanket bog Grasslands/fens Bracken Eroding blanket bog Artificial surfaces Shadow Watercourses/bodies
SM	Wet heath Dry heath Blanket bog Grasslands/fens Artificial surfaces (including exposed rocks) Shadow Watercourses/bodies

#### b. Data pre-processing

##### Generation of atmospherically corrected surface reflectance data

Each pixel in a single band of the satellite imagery was represented by the top-of-atmosphere reflectance scaled by 10,000. Hence, we divided each pixel value by 10,000 to rescale them to floating-point values 0.0 to 1.0, consistent with the range of reflectance. To correct the image for the influence of the atmosphere, we undertook the Dark Object Subtraction (DOS) method. DOS is a simple atmospheric correction method wherein the assumption is that the darkest pixels (e.g., deep water, shadow) would have zero reflectance, if not for the effects of the atmosphere on the image (Chavez, 1988). Here, the pixel values within the 10th percentile in a single band were averaged, and the result was then subtracted from all pixel values from that band. This process was repeated for all the bands of the satellite imagery, resulting in an atmospherically corrected image.

##### Variable preparation

For each study site, variables were prepared to be used as input for the modelling (Table 2). Individual atmospherically corrected image bands were included as they can provide information in the visible and NIR regions of the image. Various vegetation indices were generated as they have been found to perform well in assessing vegetation cover and are extensively used for mapping habitats and vegetation communities (Bendig et al., 2015; Bhatnagar et al., 2020; Cruz et al., 2023; Suo et al., 2019). A further variable related to Principal Component Analysis (PCA) was also developed. PCA is a technique that reduces data dimensionality by creating new, uncorrelated variables known as the Principal Components (Jolliffe, 2002). Here, the first Principal Component band, having an eigenvalue of more than 75%, was extracted as it contains most of the information from the original data (see Supplementary Information 1). Additionally, four textural variables (mean, contrast, variance and correlation) were derived based on the grey-level co-occurrence matrix (Haralick et al., 1973) of each image band. Specifically, these variables were computed over a 5 ✕ 5 neighbourhood. Previous remote sensing studies have shown that the inclusion of textural variables in the classification improves accuracy, as they can describe spatial patterns and variations of the features within a band (Barrett et al., 2016; Kattenborn et al., 2019). Finally, elevation and slope were also generated as they describe the site topography, which can be determinants of habitat distribution (Cruz et al., 2023; Scholefield et al., 2019; Zuleta et al., 2018). Overall, 33 variables were considered in the study. The description of each variable is shown in Table 2.

**Table 2:** List of variables used in the study and their descriptions

Variables		Description	Reference
Spectral	Blue band (B)	Spectral reflectance value of the blue band	
	Green band (G)	Spectral reflectance value of the green band	
	Red band (R)	Spectral reflectance value of the red band	
	Near-infrared band (NIR)	Spectral reflectance value of the near-infrared band	
	Simple Ratio (SR)	$SR= \frac{NIR}{R}$	(Jordan, 1969)
	Normalised Difference Vegetation Index (NDVI)	$NDVI= \frac{(NIR-R)}{(NIR+R)}$	(Rouse et al., 1973)
	Enhanced Vegetation Index (EVI)	$EVI=2.5*\frac{(NIR-R)}{[NIR+\left(6*R\right)-\left(7.5*B\right)+1]}$	(Huete et al., 2002)
	Normalised Difference Water Index (NDWI)	$NDWI= \frac{(G-NIR)}{(G+NIR)}$	(McFeeters, 1996)
	Modified Soil Adjusted Vegetation Index 2 (MSAVI2)	$MSAVI2=\frac{\left(2*NIR\right)+1-\surd ({2*NIR+1)}^{2}-8*\left(NIR-R\right)}{2}$	(Qi et al., 1994)
	Atmospherically Resistant Vegetation Index (ARVI)	$ARVI=\frac{(NIR-RB)}{(NIR+RB)}$	(Kaufman & Tanre, 1992)
	Green Normalized Difference Vegetation Index (GNDVI)	$GNDVI= \frac{(NIR-G)}{(NIR+G)}$	(Gitelson et al., 1996)
	RGBVI (Red Green Blue Vegetation Index)	$RGBVI=\frac{{G}^{2}-(B \times R)}{{G}^{2}+(B \times R)}$	(Bendig et al., 2015)
	Modified Green Red Vegetation Index (MGRVI)	$MGRVI=\frac{{G}^{2}-{R}^{2}}{{G}^{2}+{R}^{2}}$	(Bendig et al., 2015)
	Optimised Soil Adjusted Vegetation Index (OSAVI)	$OSAVI= \frac{(NIR-R)}{(NIR+R+0.16)}$	(Rondeaux et al., 1996)
	First Principal Component band (PC1)	The first principal component that accounts for the greatest variance in the dataset	(Jolliffe, 2002)
Textural	Textural variables	Represent spatial variations surrounding the pixel (mean, contrast, variance and correlation)	(Haralick et al., 1973)
Topographic	Elevation	Represents the height of the terrain	
	Slope	Represents the rate of change of elevation	

##### Image resampling

**Fig. 4:**
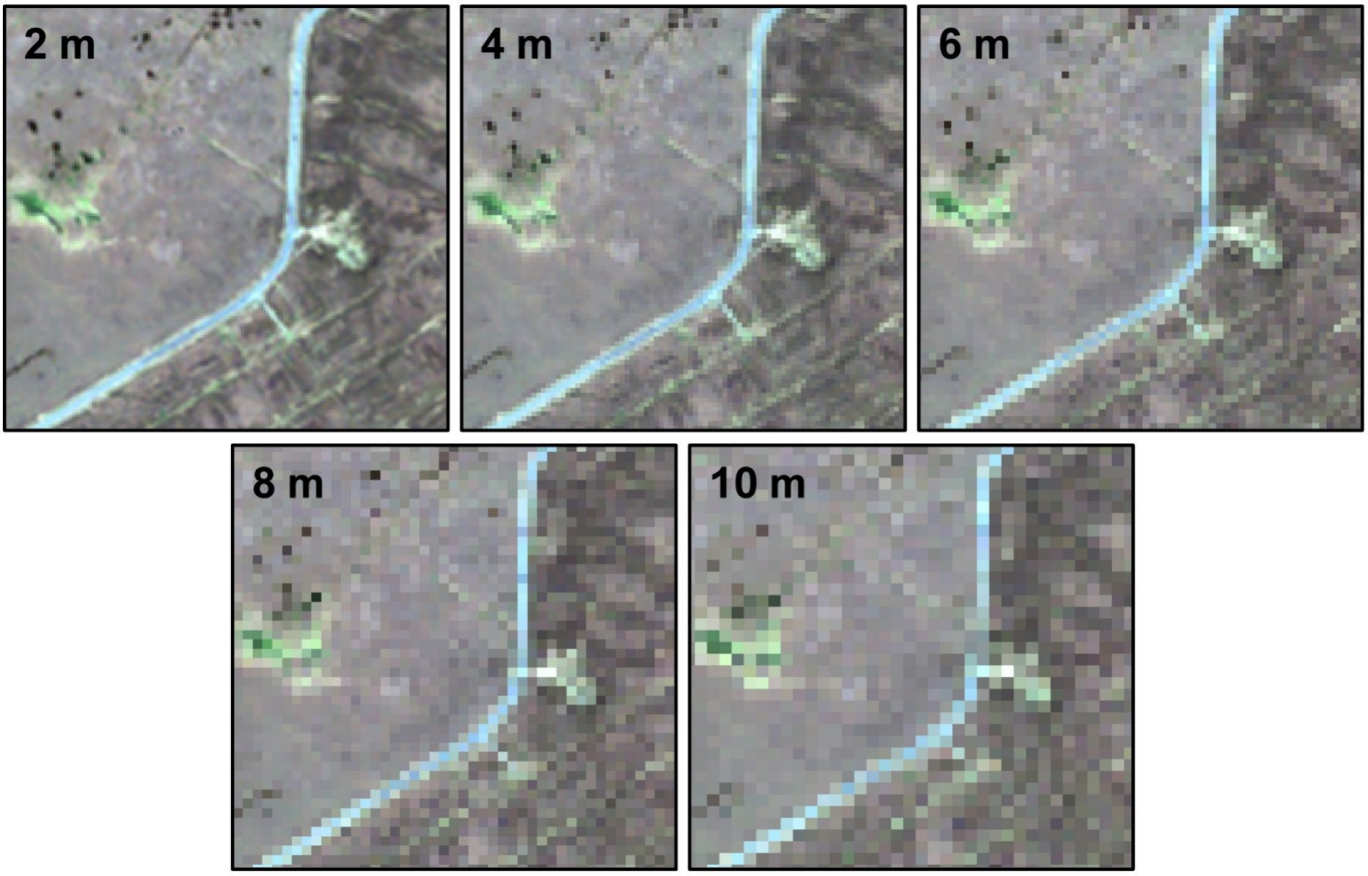
Sample images after resampling from the original 2-m resolution pixel size to 4-m, 6-m, 8-m and 10-m resolution pixel sizes

To determine the impact of spatial resolution on the classification accuracy, all variables were resampled from the original 2-m spatial resolution to 4-, 6-, 8- and 10-m resolutions (Fig. 4). This created five datasets for each site. Ten metres was selected as the lowest resampled resolution because freely accessible Sentinel-2 satellite images have this spatial resolution for the visible and NIR bands. This way, the accuracy difference between a high spatial resolution commercial satellite image and a free medium-resolution satellite image can be assessed.

The resampling technique was based on averaging the pixel values within the new pixel size. This technique, however, could result in multiple field data points falling within a single pixel of the coarser image. Hence, we reviewed their locations on the 10-m resolution image to ensure no more than one point within a pixel.

#### c. Model development and validation

The Scikit-learn Python library (Pedregosa et al., 2011) was used to implement the RF technique. This technique was applied to each dataset (i.e., variables of different spatial resolutions) to create a habitat classification model. Thus, five models were developed for each site (Table 3). Each model was evaluated using the *k*-fold cross-validation method. In this method, the points were randomly divided into *k* subsets or folds of approximately equal size. Each fold was used to evaluate the model, which was trained on the remaining *k* – 1 folds; this process was repeated until all folds were used in the validation (Berrar, 2019). Five folds were used in this study, which means that the model training and validation process was repeated five times. The average and standard error of the accuracy scores were then computed across these five folds. The final model to predict the pixel labels for the entire study site used all the points. Additionally, the relative importance of variables for each model was analysed based on Mean Decrease in Impurity (MDI) (Breiman et al., 1984).

**Table 3:** List of RF-based models developed for this study. Each model of WM and SM sites was trained on different spatial resolutions (2 m, 4 m, 6 m, 8 m and 10 m)

Spatial resolution	Models	
	WM site	SM site
2 m	WM_2m	SM_2m
4 m	WM_4m	SM_4m
6 m	WM_6m	SM_6m
8 m	WM_8m	SM_8m
10 m	WM_10m	SM_10m

#### d. Model prediction

Using the final model developed for each site, we generated two predictions: habitat predictions (crisp) and habitat probability predictions (fuzzy). The *predict()* function in Scikit-learn was used to return the predicted class for each pixel, i.e., the class with the highest probability across all the decision trees, generating a single image with row and column dimensions similar to the input satellite image. The *predict proba()* function was used to return a vector of class probabilities within the interval [0,1] for each pixel that were computed by averaging the class-predicted probabilities of all the decision trees. This latter function generates multiple images where each image represents the probabilities for a different habitat considered by the model (i.e., one image for each habitat).

To help visualise the spatial confidence of the classification associated with probabilities, we used entropy. In machine learning, entropy is defined as a measure of purity in a data set (Shannon, 1948). It is computed by:

$$E\left(p\right)=-\sum\nolimits_{n=1}^cp_i{log}_2p_i,$$

where $p$ is the probabilities obtained from the fuzzy classification. The value of entropy is inversely proportional to the confidence in the prediction. In other words, the entropy value is low for high-confidence predictions (peaky probability distribution), and the entropy value is high for low-confidence predictions (flat probability distribution). For example, if two pixels have probability predictions of (0.05, 0.90, 0.05) and (0.35, 0.40, 0.25), it can be considered that the first pixel was predicted with higher confidence, i.e., having a lower entropy value, than the second pixel.

## Results

### Effect of spatial resolution on mapping upland habitats

Table 4 summarises the results obtained by the 5-fold cross-validation method for WM and SM models trained at five different spatial resolutions. Overall, the models trained using a higher spatial resolution dataset generally achieved better performance. For both sites, the models trained on 2-m resolution datasets had the highest mean accuracy (WM_2m: 80.34% and SM_2m: 79.64%), followed by models trained on 4-m resolution datasets (WM_4m and SM_4m) with an accuracy of 77–79%. Furthermore, there was a decrease in accuracy of about 4–7% when the coarser 10-m resolution datasets were used for model training compared to when the 2-m resolution datasets were used. However, this trend of higher spatial resolution datasets leading to better classification accuracy was not always the case. For example, the coarser resolution-based WM_8m model achieved a higher accuracy (75.94%) than the WM_6m model (73.71%). Similarly, the SM_10m model performed better than the SM_8m model (75.44% vs. 73.83%).

**Table 4:** 5-fold cross-validation mean accuracy and standard error results for WM and SM models. Descriptions of the models are presented in Table 3

Wicklow Mountains site			Slieve Mish Mountains site		
Models	Mean accuracy (%)	Standard error	Models	Mean accuracy (%)	Standard error
WM_2m	80.34	3.78	SM_2m	79.64	5.43
WM_4m	77.47	3.21	SM_4m	78.78	5.15
WM_6m	73.71	3.92	SM_6m	77.81	6.34
WM_8m	75.94	2.10	SM_8m	73.83	6.25
WM_10m	73.55	1.95	SM_10m	75.44	7.61

**Fig. 5:**
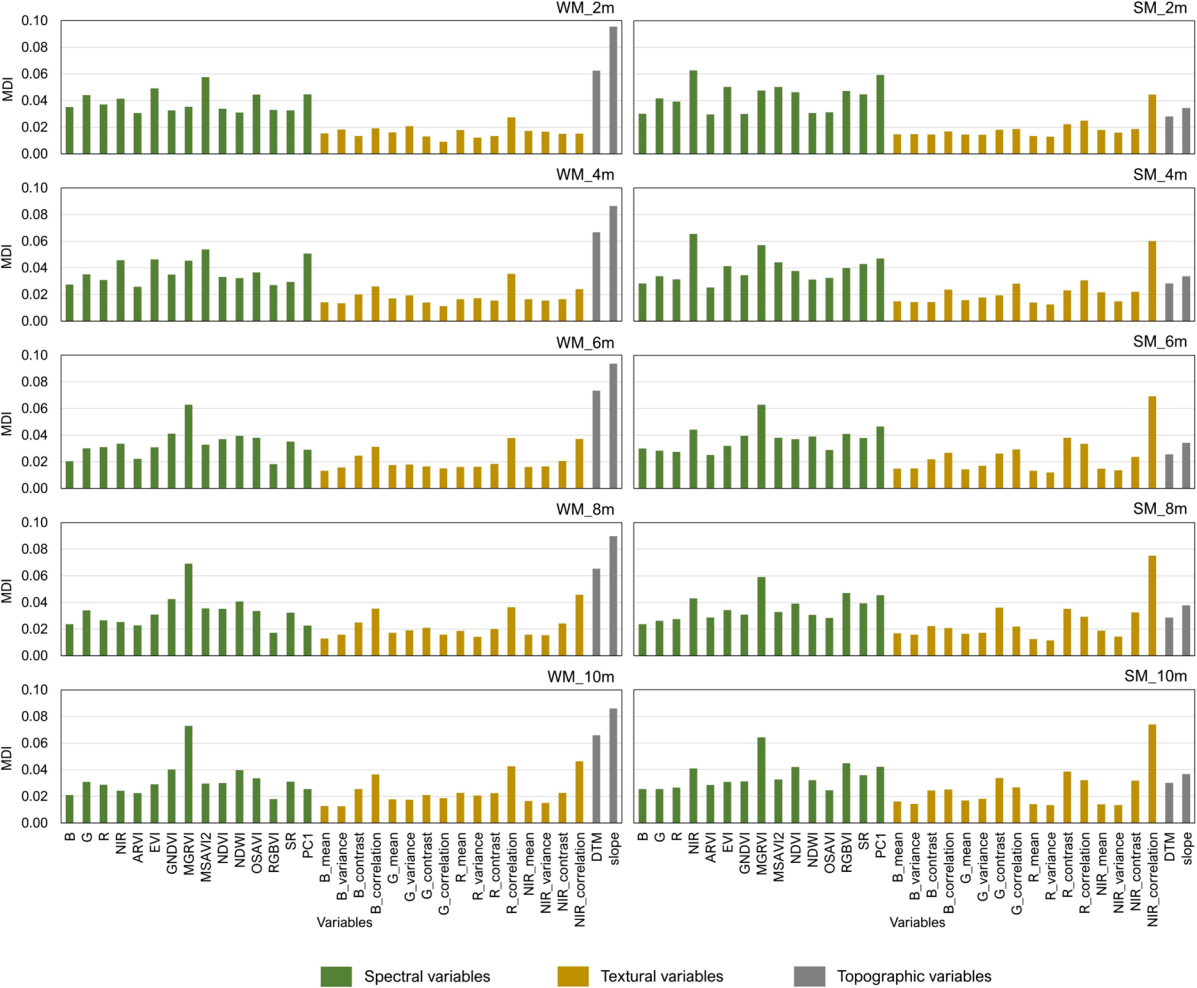
Variable importance scores for WM and SM models at different spatial resolutions. The x-axis displays the input variables analysed in the study, while the y-axis represents the variable importance score calculated using MDI. Variable descriptions can be found in Table 2

The contributions of each input variable to the model predictions are summarised in Fig. 5. In general, spectral variables consistently showed higher importance for both sites, while textural variables presented lower importance values. It can be observed that the importance of variables varied depending on the spatial resolution of the datasets used in the modelling. For example, the importance of textural variables increased gradually as the spatial resolution coarsened. Whereas for spectral variables, their importance values slightly decreased with lower spatial resolutions. Elevation and slope variables showed almost similar importance values across spatial resolutions.

Models for each site exhibited different important variables. The elevation and slope, representing the topography of the site, were often ranked as the most important variables in relation to the WM models. Conversely, MGRVI and NIR_correlation were the most important variables in the majority of the SM models (Fig. 5).

**Fig. 6:**
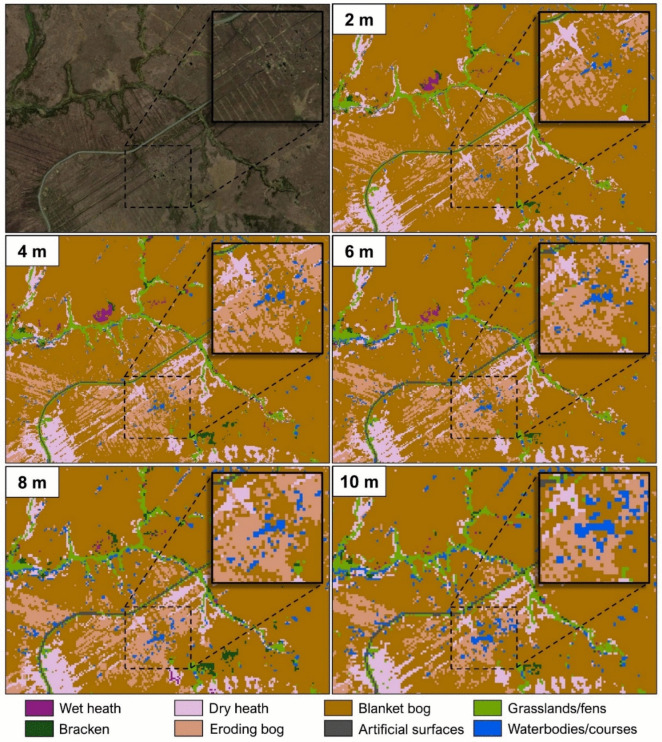
Crisp classification results over a portion of the WM site (top left) based on five different spatial resolutions (2 m, 4 m, 6 m, 8 m and 10 m). The zoomed-in sections illustrate the increasing size of mapped waterbodies with lower spatial resolution datasets

Figure 6 shows the crisp RF-based classified images of a part of the WM site to compare the impact of using different spatial resolution datasets on model output. Overall, classification results across the five spatial resolutions demonstrated comparable patterns of habitat distributions with greater spatial detail in the 2-m classified image. These details, however, differ depending on the spatial resolution used. For example, the size of mapped waterbodies within the zoomed-in sections of Fig. 6 increased when using lower spatial resolutions. A similar trend was also observed for patches of eroding blanket bogs.

**Fig. 7:**
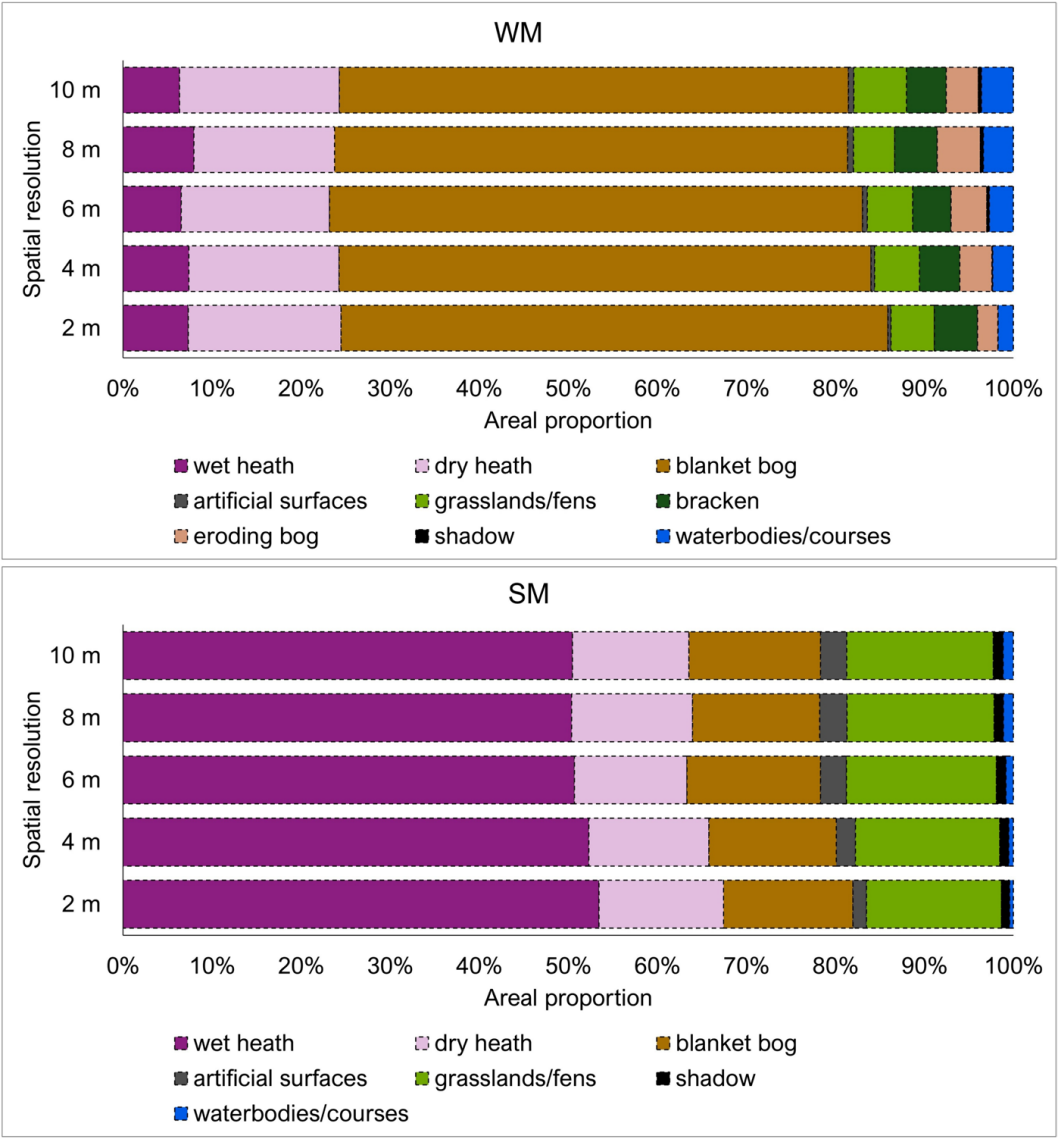
Areal proportion for each habitat computed from the model classification results for WM and SM sites

Figure 7 displays the areal proportions of habitats as classified using different resolutions for the WM and SM sites. No significant differences were observed in the areal proportions between the different habitats. However, it can be observed that habitats with the smallest proportions generally increased in size with lower spatial resolutions.

### Model predictions

**Fig. 8:**
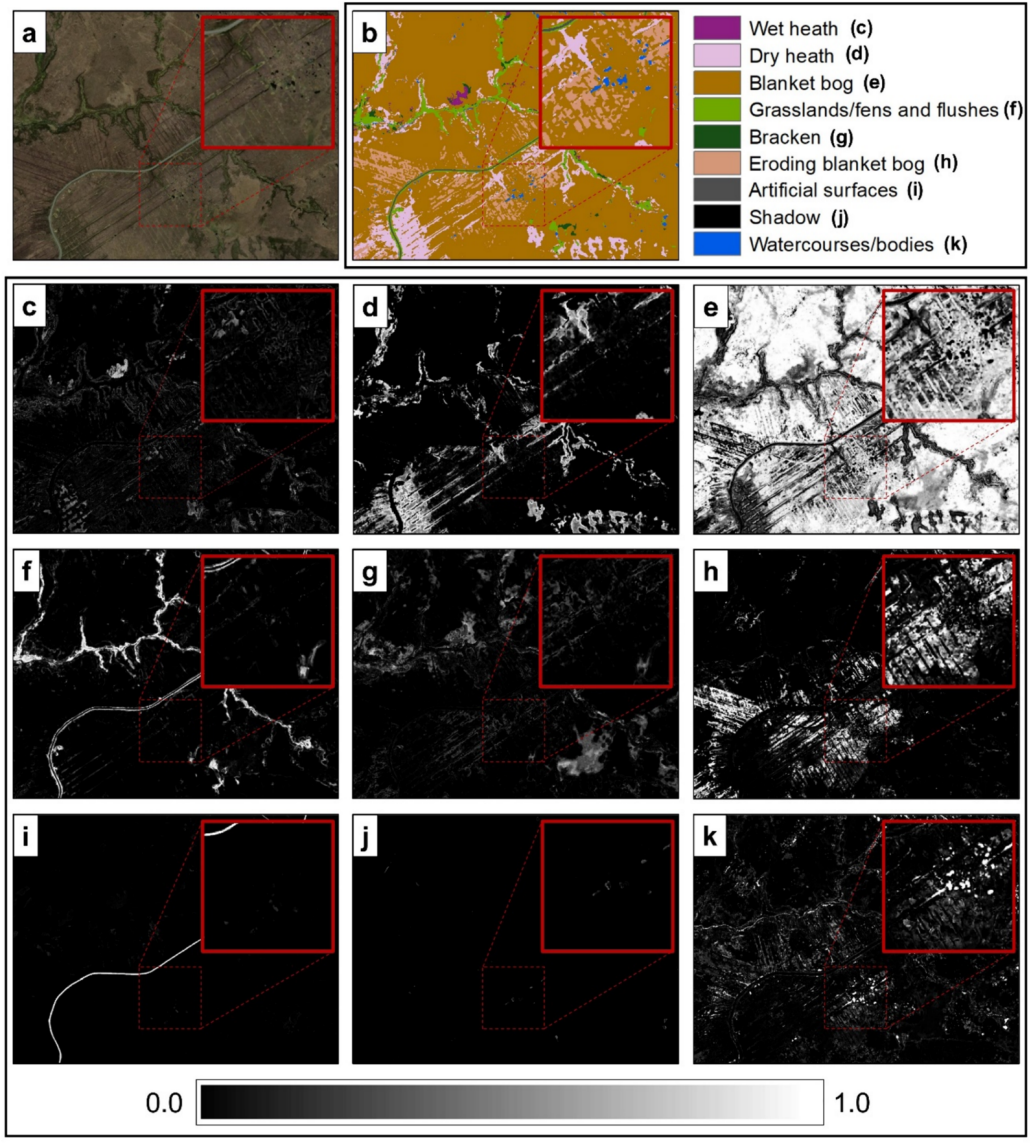
Basemap of a portion of the WM site (**a**) and the corresponding crisp (**b**) and fuzzy (**c-k**) classification results. The colour scale below represents the probability of each habitat being present in every pixel

Figure 8 shows the crisp and fuzzy classification results over a portion of the WM site (Fig. 8a). In the crisp classification result (Fig. 8b), each pixel was characterised by a single habitat type. In contrast, the fuzzy classification results (Fig. 8c–k) represented the probability of each habitat being present on each pixel, i.e., there was one greyscale image for every habitat type. Hence, the number of output fuzzy images corresponds to the number of habitats considered in the modelling. The white pixels in the image indicate areas where a particular habitat was predicted to be 100% present in that pixel. For example, blanket bog (Fig. 8e) and grasslands/fens (Fig. 8f) were some of the habitats that were distinctly separated from their surroundings. In contrast, black pixels represent those areas where a habitat was predicted with 0% probability, indicating its absence in that pixel. Grey pixels, therefore, denote predictions that fall between 0.0 and 1.0. These predictions could suggest areas of mixed habitats. The total of all the probabilities in a pixel adds up to 1.0. The complete crisp and fuzzy classification results for WM and SM sites can be found in Supplementary Information 2.

### Distribution and extent of upland habitats

**Fig. 9:**
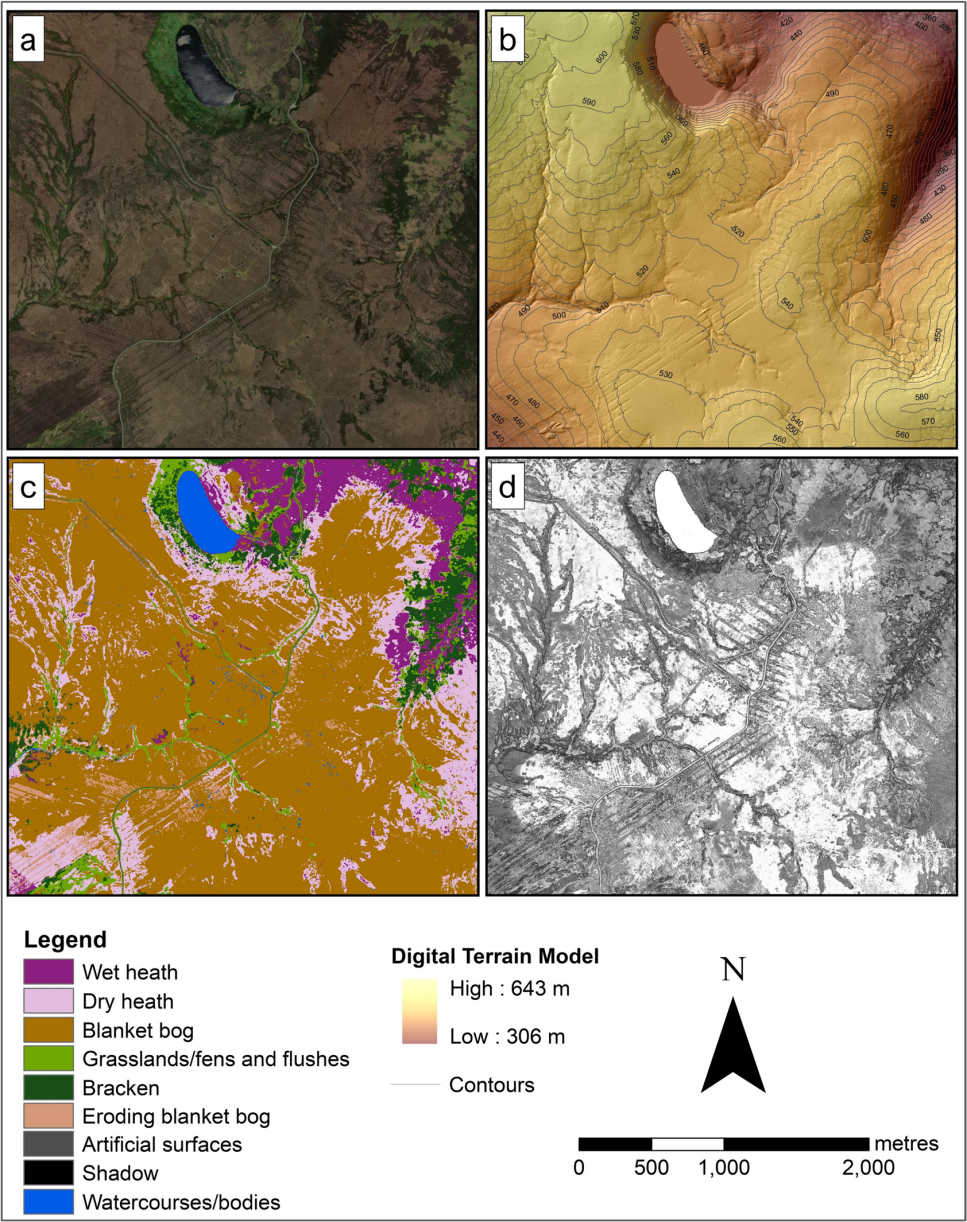
Basemap (**a**) and the DTM (**b**) of the WM site, and the corresponding crisp classification result (**c**) and entropy image (**d**) for the 2-m resolution model. The colours in (**d**) represent the level of certainty: lighter colours being more certain and darker colours being less certain

The WM site (Fig. 9a) was dominated by blanket bogs, covering almost 60% of the site. This habitat was most extensive on a relatively flat or gently sloping higher altitude part of the site (Fig. 9b and c). Large areas of wet heath and dry heath were found on the hillsides, where the terrain was characterised by steep slopes (Fig. 9b and c). In these areas, mosaics of dense bracken and grasslands/fens were also present. Eroding blanket bog areas were located near the roads (i.e., classified as artificial surfaces) and often occurred in straight parallel lines, intermixed with dry heath. The site was also characterised by a big lake in the north and several small bog pools around the centre of the map; both were classified as waterbodies/courses (Fig. 9c).

The colour scale of the entropy image (Fig. 9d) indicates the level of certainty in the classification, i.e., light pixels being more confident and dark pixels being less confident. The areas mapped as blanket bogs and as other common features that are spectrally distinct, such as waterbodies and roads, were classified with high certainty, as observed by the light colour on the map (Fig. 9d). However, most wet heath or dry heath areas were classified with less certainty.

**Fig. 10:**
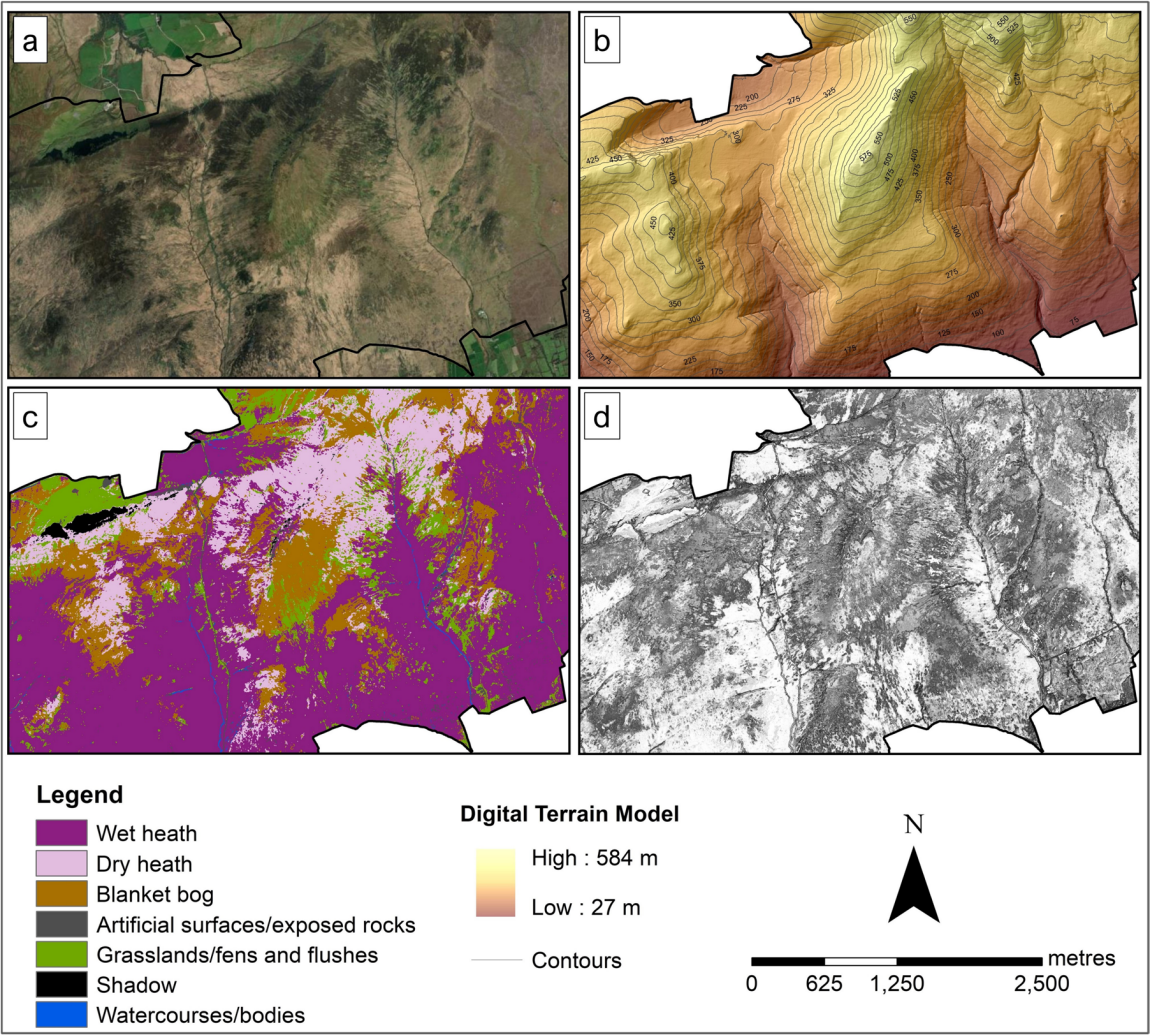
Maps showing the basemap (**a**) and the DTM (**b**) of SM, and the corresponding crisp classification result (**c**) and entropy image (**d**) for the 2-m resolution model. The colours in (**d**) represent the level of certainty: lighter colours being more certain and darker colours being less certain

The SM site (Fig. 10a) was covered by about 50% of wet heath. This habitat was mainly located at lower altitudes of the site and where the slope was less steep. As the altitude increases and the slope gradually becomes steep, wet heath habitat transitioned into mosaics of blanket bogs, dry heath and grasslands/fens (Fig. 10b and c). Patches of exposed rocks/scree were scattered across the site. The site was also crossed by narrow watercourses. Shadow was recorded in the north-western part of the SM site due to the quite steep slope obscuring the actual habitat. The entropy image (Fig. 10d) showed that the dominant cover wet heath areas were classified with high certainty, i.e., low entropy. In contrast, dry heath and blanket bog were the areas mapped with less certainty, illustrating an ambiguity in the prediction, possibly due to these habitats occurring in mosaics.

## Discussion

This study demonstrates the potential of using high-resolution Pleiades satellite imagery and RF machine learning to accurately map upland habitats in two designated upland sites. The spatial distribution maps produced using the developed RF models provide essential information on the extent and location of protected habitats, as well as on the degraded versions of blanket bogs.

The methodology was designed to evaluate the impact of varying image spatial resolutions on classification accuracy. Results showed that using finer spatial resolution data generally leads to improved classification accuracy, consistent with other satellite-based remote sensing studies (Boyle et al., 2014; Dorji & Fearns, 2017; J. Fisher et al., 2017). The observed differences between the area of each habitat, depending on what spatial resolution was used in the modelling (see Fig. 7), could be explained by the lower spatial resolution imagery overestimating and underestimating different habitats. In this study, habitats that occurred as small patches, such as bog pools, were highly influenced by an overestimation of area, while dominant habitats, such as blanket bogs and heath, were affected by an underestimation of area when a lower spatial resolution image was used (see Fig. 6 and Fig. 7). This finding has implications when using freely available satellite imagery, such as Sentinel-2 and Landsat-8, for assessing highly heterogeneous or fragmented landscapes. A coarse-resolution image may under- or overestimate the actual size of different habitats.

While the results showed only slight differences in the computed area when mapping with different spatial resolutions (Δ_ave_ = 14.7 hectares between the 2-m and 10-m resolution images), this may lead to a more significant difference if the mapping is conducted on a regional scale. This variation in areas may lead to inaccurate assessments and reporting of the actual habitat status. For example, Hernando et al. (2017) demonstrated that the observed spatial patterns (i.e., connectivity and fragmentation) in forest cover maps varied based on the spatial resolutions of the data used, leading to different conservation status assessments of those maps. Therefore, it is important to consider using an image with a fixed spatial resolution when monitoring habitats with remote sensing to ensure reliability in reporting spatial extents and accurately measuring the change over time. Reporting the spatial resolution of the data used in the analysis is also crucial.

The study also compared two RF mapping techniques in classifying upland habitats: crisp and fuzzy. Both techniques can map the general distribution of upland habitats at WM and SM sites. In the crisp classification technique, the distribution of upland habitats is presented as a single map. However, this technique (i.e., assigning one habitat per pixel) may oversimplify the typical complex distributions of upland habitats observed in the field, especially when using images of lower spatial resolutions (Fig. 6). In the fuzzy classification technique, the distribution of habitats is presented in multiple maps, providing rich information (probabilities of habitat occurrence per pixel) about the habitat composition and patterns (Fig. 8c-k). The technique can also be applied to detect transition zones between habitats. These zones are observed as the spatial transition in the probability of a habitat from high to low, and in the same area, an inverse pattern of probability change (i.e., from low to high) is exhibited by another habitat (Feilhauer et al., 2021). The study of de Klerk et al. (2018) also used probability maps to further describe and categorise transition zones—depending on their widths—as sharp, moderate and slow transitions. While these zones can be mapped as separate classes using the crisp technique, this may result in many classes, making it difficult to interpret the map (Feilhauer et al., 2021).

Moreover, the study demonstrated the use of entropy images as a measure to estimate confidence predictions. Previous studies have also proposed other measures, such as confusion indices and RGB colour blend images, for estimating confidence in predictions generated from fuzzy classification (Duff et al., 2014; Feilhauer et al., 2021; Zlinszky & Kania, 2016). Understanding a set of fuzzy maps may not be straightforward, especially for non-specialist users; therefore, most fuzzy maps are converted to a single crisp classified map (Zlinszky & Kania, 2016). While conservation managers may prefer using crisp classification maps because they are generally easy to interpret, they can also benefit from entropy images. These images can be used to identify areas on the crisp classified map that are most likely occupied by mosaics of habitats and not just by a single habitat that the crisp classified map indicates. This could suggest that using entropy images can have a more practical significance when mapping with freely accessible satellite images (e.g., Landsat-8 and Sentinel-2), as their coarse spatial resolutions would mean that a single pixel most likely covers more than one habitat. Also, areas on the map with higher entropy values are considered less accurate than areas with lower entropy values; further assessment of these less accurate areas can be recommended especially if they were previously assessed with lower entropy values, i.e., considered highly accurate. Therefore, this finding suggests that presenting habitat distribution with a crisp classified map can be supplemented with its corresponding entropy image as the latter provides additional information about the confidence in predictions.

**Fig. 11:**
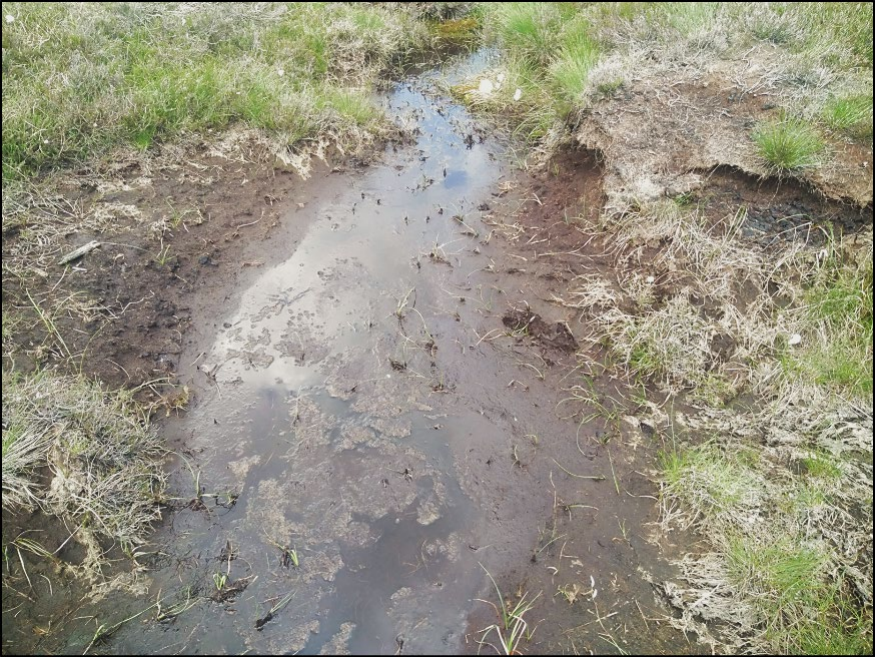
Eroded peat surface in the WM site. (Photo: P. Perrin, July 2021)

The results highlighted that small or narrow patches were clearly mapped in higher spatial resolution imagery. Detecting small but important features on the map may help better assess the habitat condition. For example, eroding blanket bogs (Fig. 11), which are often characterised by a network of channels occurring in linear patterns (Fossitt, 2000), were visibly delineated on the WM_2m map (Fig. 9c). Missed detection of small-sized degradation may lead to false conclusions on the habitat status. Moreover, the information on the locations of these eroding bogs (i.e., mapped closer to the road) may indicate that bogs closer to the road are more vulnerable to anthropogenic activities, such as drainage and turf-cutting, than those located far from the road. In the report of Perrin et al. (2017), a lot of the eroding bogs at the WM site is the result of peat extraction by hand and using sausage machines. Therefore, creating detailed and accurate maps has an important implication for conservation and can be used to propose recommendations, e.g., bogs closer to the road require special attention for protection as the soil carbon loss in these areas may be significant.

Overall, while there is a high cost involved in acquiring higher-resolution satellite data, the detailed information it can provide is of utmost importance in habitat conservation, particularly in spatially extensive, heterogeneously complex landscapes. The crisp and fuzzy mapping approaches presented here can also be used to represent the natural complexity of the spatial distribution of upland habitats. Future studies can explore applying these approaches for mapping uplands more extensively as well as mapping dynamic habitats, such as coastal areas, and those with spectrally similar vegetation communities, such as grasslands.

## Conclusion

This study used high-resolution satellite imagery and RF machine learning to map upland habitats. Using higher spatial resolution imagery generally improves mapping accuracy. In the two upland sites we studied, the highest accuracy maps were obtained (WM site – 80.34% and SM site – 79.64%) with the 2-m resolution datasets. These maps provide information on the spatial distribution of habitats in great detail. Coarser spatial resolution datasets, however, resulted in a reduction of the accuracy and a slight overestimation of area for narrow and small-sized habitats. Therefore, a higher spatial resolution dataset is preferred if mapping habitats in a more heterogeneous and diverse landscape. The study also demonstrated the use of crisp and fuzzy classification techniques in mapping upland habitats. Crisp classification results in a single habitat map, which is relatively easy to interpret. Fuzzy classification delivers probability maps for each habitat considered in the modelling. While these maps may be more difficult to interpret, they can represent the typical complex mosaics and gradual transitions of upland habitats as observed in the field. They can also be used to describe spatial confidence in the classification through computing the entropy. Using fuzzy classified maps has the potential to improve our understanding of nature’s fuzzy patterns.

## Data Availability

The data supporting this study’s findings are available from the corresponding author, Charmaine Cruz (cruzc@tcd.ie), upon reasonable request.

## Declarations

All authors have read, understood, and have complied as applicable with the statement on "Ethical responsibilities of Authors" as found in the Instructions for Authors.
